# Diffusion tensor imaging along the perivascular space in healthy young adults is associated with decreased sustained attention: a preliminary study

**DOI:** 10.1007/s11682-026-01112-2

**Published:** 2026-02-25

**Authors:** Gergely Darnai, Ákos Arató, Barnabás Dudás, Anna Tímea Szente, Gábor Perlaki, Gergely Orsi, Szilvia Anett Nagy, József Janszky

**Affiliations:** 1 Department of Neurology, Medical School, University of Pécs, Pécs, Hungary; 2 Department of Behavioural Sciences, Medical School, University of Pécs, H-7624, Pécs, Szigeti street 12, Pécs, Hungary; 3 Clinical and Cognitive Neuroscience Research Group, Szentágothai Research Centre, University of Pécs, Pécs, Hungary; 4 HUN-REN-PTE Clinical Neuroscience MR Research Group, Pécs, Hungary; 5 Pécs Diagnostic Centre, Pécs, Hungary; 6 Department of Neurosurgery, Medical School, University of Pécs, Pécs, Hungary; 7 Neurobiology of Stress Research Group, Szentágothai Research Centre, University of Pécs, Pécs, Hungary; 8 Department of Medical Imaging, Faculty of Health Sciences, University of Pécs, Pécs, 7621 Hungary

**Keywords:** DTI-ALPS, ALPS index, Diffusion tensor imaging, Psychomotor vigilance task, Time-on-task effect, Intracranial fluid dynamics

## Abstract

Diffusion Tensor Imaging Along the Perivascular Space (DTI-ALPS) is a noninvasive method often used to assess and quantify diffusivity along the perivascular space in predefined regions of the brain. Although it is frequently applied in clinical populations, its relationship with cognitive performance in healthy individuals remains poorly understood. This study examined whether DTI-ALPS index (hereafter ALPS index) values are associated with performance decrement during a sustained attention task in healthy young adults. Forty-two healthy right-handed participants underwent DTI scanning and performed a 25-minute psychomotor vigilance task (containing 5-minute long blocks) in an MRI environment. ALPS indices were computed separately for the left and right hemispheres. Intraclass correlation coefficients between the observers were calculated to assess interrater reliability. Performance changes during the vigilance task were quantified by mean reaction time (RT) changes between the first and last 5-minute-long blocks (B5–B1 difference) and RT slope across the 5-minute-long task blocks. The observer agreement for ALPS index was acceptable to excellent (0.754–0.902). Behavioral analyses confirmed a significant increase in RTs across the blocks (*p* < 0.001). Both left and right ALPS indices were significantly and inversely associated with RT increase (left: *p* = 0.002; right: *p* = 0.003) and RT slope (left: *p* = 0.003; right: *p* = 0.004), indicating that higher ALPS index values predicted greater resilience to cognitive fatigue. Our findings suggest that ALPS index is correlated with sustained attention performance in healthy individuals.

## Introduction

Diffusion Tensor Imaging Along the Perivascular Space (DTI-ALPS) has emerged as a noninvasive technique for assessing diffusivity along the perivascular space (PVS). This method quantifies diffusion along the PVS as the ratio of the mean diffusivity measured in the x-/PVS-direction (over the projection and association fibers) to the mean of the diffusivity perpendicular to both the x-direction and the projection fibers as well as the diffusivity perpendicular to both the x-direction and the association fibers, at the level of upper part of lateral ventricle body (Taoka et al., 2017). DTI-ALPS has been shown to be highly reproducible and scanner differences did not affect DTI-ALPS index (hereafter ALPS index) values, when the basic imaging conditions were matched (Taoka et al., 2022). Several previous studies speculated that the ALPS index indicates the level of functionality and the extent of impairment of the glymphatic system, which regulates directional interstitial fluid movement and waste clearance (Taoka et al., 2017). It is important to highlight that although several previous studies interpreted ALPS index as uniquely corresponding to the function of the glymphatic system, the interpretation should be much more cautious because the relationship between the ALPS index and human glymphatic function has not been rigorously validated by pathophysiological studies (Taoka et al., 2022). Other problems with the ALPS index are (i) the subjectivity and arbitrariness of manual region of interest (ROI) placement, (ii) the ROIs may be affected by partial volume effects of the nearby fiber tracts, and (iii) the ROIs for calculating the ALPS index include not only the PVS, but also the surrounding white matter (it is not possible to evaluate only the diffusivity of the PVS) (Ringstad, 2024; Taoka et al., 2024).

Because the ALPS index is given by a simple formula and can be calculated from retrospective data, it has been reported in many diseases, pathologies, and conditions. It has been repeatedly found to decrease in Alzheimer’s disease (Kamagata et al., 2022; Ota et al., 2022; Steward et al., 2021; Zhang et al., 2024; Zhong et al., 2023), Parkinson’s disease, mild cognitive impairment (Bae et al., 2023; Chen et al., 2021; He et al., 2023; Ma et al., 2021; McKnight et al., 2021; Meng et al., 2024; Shen et al., 2022; Si et al., 2022), small vessel diseases (Tang et al., 2022; Tian et al., 2023; Xu et al., 2022), normal pressure hydrocephalus (Charalampos et al., 2023; Eide et al., 2022; Yokota et al., 2019), traumatic brain injury (Dai et al., 2023; Yang et al., 2024; Yuichi et al., 2023), demyelinating diseases (Carotenuto et al., 2022) and sleep disorders (Gumeler et al., 2023; Roy et al., 2022; Siow et al., 2022). Some of these studies investigated whether ALPS index is related to cognitive performance. These studies mostly assessed general cognitive performance using the Mini Mental State Examination (Charalampos et al., 2023; Chen et al., 2021; Ma et al., 2021; Steward et al., 2021; Xu et al., 2022; Zhong et al., 2023) or Montreal Cognitive Assessment (Tang et al., 2022; Xu et al., 2022; Yuichi et al., 2023; Zhong et al., 2023) and revealed a positive association between performance and the ALPS index. Some articles focused on concrete cognitive modules such as attention and working memory (He et al., 2023; Tang et al., 2022; Zhang et al., 2024), long-term memory (He et al., 2023), verbal performance (He et al., 2023; Tang et al., 2022; Zhang et al., 2024) and learning skills (Tang et al., 2022). Similar to general cognitive scores, these studies also explored positive association between cognitive performance and ALPS index. Lastly, two studies showed that the ALPS index is not only related to actual cognitive scores, but can also reliably predict cognitive decline (He et al., 2023; Si et al., 2022). To the best of our knowledge, only one study has investigated the relationship between cognition and ALPS index in healthy individuals. This study focused primarily on the effect of age; thus, the age range was wide, between 10 and 80 years. They found an age-dependent decreasing trend of ALPS index and ALPS index was related to mental manipulation score (that is a subscore of Cognitive Abilities Screening Instrument) when the whole sample was included and to short-term memory in 50 + healthy elderly participants (the models were adjusted for age in both cases) (Hsiao et al., 2023).

In 2023 our research group conducted a study in which we intended to explore the brain mechanisms behind visual attention and mental fatigue. The study included diffusion tensor imaging (DTI) and a prolonged 25-minute-long psychomotor vigilance task (PVT) that allowed us to investigate whether the ALPS index is associated with the extent of performance decrease during a sustained attention task. The ALPS indices were calculated separately for both hemispheres. Since the manual ROI placement may add subjectivity to evaluations, three observers were involved and intraclass correlation coefficients were calculated between them. We expected that similar to previous studies reporting a higher ALPS index in association with better cognitive performance, ALPS index would be negatively correlated with performance decrease during the PVT. To date, no study has directly examined the association between the ALPS index and sustained attention in either clinical or healthy samples, and most of the limited work on cognition has relied on questionnaire-based measures rather than sensitive, performance-based computerized tasks such as the one used in the present pilot study.

## Methods

### Participants

Our sample comprised of 42 participants (18 males; mean age: 22.5 ± 2.0 years). Given the sensitivity of the Psychomotor Vigilance Task (PVT) to sleep propensity, sleep quality and sleep time were an inclusion criterion. Participants were instructed to maintain regular sleep schedules (sleep at least 6 h prior) and completed a sleep diary to verify compliance. Subjects who slept for fewer than six hours before the day of the MRI were not included in the investigation. We also assessed subjective sleep quality using a seven-point Likert scale. Participants rated the quality of their sleep on the previous night on the scale from 1 to 7, where 1 indicated extremely poor sleep and 7 indicated extremely good sleep. Based on these ratings (median = 6; min-max. = 3–7), our participants generally reported adequate sleep quality on the night before the assessment. As mental fatigue is also sensitive to daytime sleepiness (Thomann et al., 2014), we used the Epworth Sleepiness Scale (ESS) (Johns, 1991) to control for its effect. To minimize the effects of the subjects’ circadian rhythms (A Van Dongen & Dinges, 2000), all measurements were taken between 1:00 p.m. and 5:00 p.m. Additionally, participants were asked to abstain from alcohol and caffeine consumption for 24 h prior to the study.

A small fee was paid to each participant as compensation for their time and efforts. The subjects had right-hand dominance according to the Edinburgh Handedness Inventory (Oldfield, 1971). All participants were informed about the study, and all provided written informed consent. The study was approved by the National Medical Research Council (registration number: 6843- 5/2021/EÜIG). All procedures performed in this study were in accordance with the 1964 Declaration of Helsinki.

### Stimuli and experimental design

The PVT was administered in the scanner using Presentation software (Neurobehavioral Systems, Inc., Berkeley, CA, USA). The stimuli were presented via an MRI-compatible LCD screen with a 1920 × 1200 resolution (BOLDscreen 24 LCD for fMRI, Cambridge Research Systems Ltd, Rochester, United Kingdom). The trials started with a white fixation cross at the center of the screen. After an interstimulus interval (which varied between 2 and 20 s in a pseudorandom range) a blue circle appeared until a response was made or 2 s elapsed. The participants were instructed to press a button as soon as the blue circle appeared. Right before the task, five practice trials were given to the participants. The experiment consisted of five 5-minute-long blocks of trials (25 min in total): B1, B2, B3, B4, and B5. After the 25-minute PVT, imaging data were acquired to assess diffusion along the PVS, with participants not performing any additional task.

### Performance measures

Firstly, as a measure of level of performance, we extracted mean reaction time (RT) for each block for each participant. To avoid behavioural findings driven by lapses we removed potential lapses (RTs > 500 ms) from the dataset. To assess performance change, the time-on-task (TOT) effect was used, which refers to a progressive decrement of performance across the duration of the cognitive task. In our case the difference in mean RTs between B5 and B1 was first obtained (B5_minus_B1). Although this is a commonly used method, as this index completely neglects the performance in the intermediate blocks (B2-B4), regression slopes were also calculated for each participant based on block-specific mean RTs (RT_slope). The two indicators were used separately in statistical analyses.

### Imaging data acquisition

All imaging data were acquired on a Siemens Prisma 3T MRI scanner with a 64-channel head-neck coil. A 2D diffusion-weighted spin-echo EPI sequence was used for diffusion tensor imaging measurements (TR/TE = 8000/69 ms; 70 axial slices; slice thickness = 2 mm; no interslice gap; FOV=256 × 256 mm^2^; matrix size=128 × 128; phase-encoding direction = AP; diffusion gradients were applied in 64 directions with a b-value of 1000 s/mm^2^ and 11 volumes were collected with no diffusion gradients applied; bandwidth = 1562 Hz/pixel; 3 volumes of b = 0 s/mm^2^ were also collected with an opposite phase-encoding direction).

### Diffusion data processing and DTI-ALPS index calculations

Susceptibility-induced off-resonance field and undistorted b = 0 s/mm^2^ (b_0_) images were calculated by FSL’s topup from the two b_0_ images acquired with opposite phase encoding directions. After creating a binary brain mask for diffusion data by running FSL’s BET on the average of the two undistorted b_0_ images, susceptibility-induced distortions, eddy current-induced distortions and subject movements were corrected as well as outlier slices with an average intensity at least 3 standard deviations lower than expected were detected and replaced with Gaussian Process prediction using the CUDA version of FSL’s eddy (eddy_cuda 10.2). Diffusion tensor model was fitted on the preprocessed data using FSL’s dtifit command. The calculated FA map of each subject was linearly registered to the 1 mm JHU-ICBM-FA template supplied by FSL using 6 degrees-of-freedom. The resulting transformation matrix was applied to the diffusion tensor using the vecreg command line tool of FSL and diffusivity maps in the x-(left-right), y-(anterior-posterior) and z-(inferior-superior) directions were extracted from the resampled tensor. For the calculation of ALPS indices, 4 spherical ROIs (each having a volume of 123 mm^3^) were placed in the projection and association fibers for both the left- and right-hemispheres at the level of upper part of lateral ventricle body in accordance with the guidelines of Tatekawa et al. (2023). This method (i.e. using vecreg function of FSL to register tensor data into standard space combined with manual ROI placement) showed good-to-excellent inter-rater reliability in our previous study (Perlaki et al., 2025), Tatekawa et al., (2023) also reported good-to-excellent intra- and inter-reliabilities for this method. To minimise bias arising from the manual placement of ROIs, three independent observers placed the ROIs for ALPS calculations. The mean DTI ALPS-index was calculated separately for the left and right hemispheres according to the following:

ALPS_index_=(Dxx, projection + Dxx, association)/(Dyy, projection + Dzz, association),

where Dxx, projection is the diffusivity along the x-direction averaged over the spherical ROI for projection fibers, Dxx, association is the x-axis diffusivity averaged over the ROI for association fibers, Dyy, projection is the y-axis diffusivity averaged over the ROI for projection fibers, while Dzz, association is the z-axis diffusivity averaged over the ROI for association fibers.

### Statistical analyses

Behavioral data were analyzed using IBM SPSS Statistics for Windows, version 25.0 (IBM Corp., Armonk, NY, USA). Intraclass correlation coefficients (ICCs) were used to assess the consistency (i.e. systematic differences are irrelevant) and absolute agreement (i.e. systematic differences are relevant) between the ALPS indices by the three observers. Two-way mixed model was selected, and both single- and average measures ICCs were obtained. As a rule of thumb, ICC values were classified as excellent (≥ 0.9), good (0.9 > ICC ≥ 0.8), and acceptable (0.8 > ICC ≥ 0.7).

To test the TOT effect, behavioral performance data were subjected to repeated-measures ANOVA with Block (i.e., the five experimental blocks) as a within-subject factor. The assumptions of ANOVA were satisfied, as judged by testing for outliers at any level of the within-subjects factor, normal distribution of variables for each level of the within-subjects factor, and sphericity. Post hoc analysis with Bonferroni adjustment was also conducted. *P* < 0.05 was used as the cutoff for significance. Effect sizes were assessed using partial eta squared (η^2^_p_).

Multiple linear regression models were used to explore the relationships between performance change (B5_minus_B1 and RT_slope) and ALPS indexes (left and right, respectively). The B5_minus_B1 and RT_slope served as dependent variables, while ALPS indices, sex, age and ESS scores served as independent variables in the models. To test whether head motion (both absolute and relative) and the percentage of replaced slices affected the regression results, QC metrics were included as covariates in the regression models. As no significant effects were found, these metrics were subsequently removed from the regression analyses. Sex, age and were included in the statistical models because they were found to be related to both ALPS index (Hsiao et al., 2023; Ozsahin et al., 2024) and TOT (Golaszewski et al., 2021). ESS scores were also used as a covariate since daytime sleepiness is found to be related to ToT as well (Thomann et al., 2014). The assumptions of multiple linear regression were satisfied as judged by checking for independence of residuals, linearity, homoscedasticity, multicollinearity, outliers, and normality of the residues. *P* < 0.05 was used as the cutoff for significance. The Benjamini-Hochberg correction was used to adjust the P-value to compensate for the false-discovery-rate across multiple comparisons.

## Results

The absolute agreement and consistency among the observers were acceptable and good, respectively, for both versions of the ALPS index (i.e. left and right) when using the single-measures ICCs and excellent when using the average-measures ones (Table 1). Therefore, the ALPS indices used in subsequent analyses were computed as the mean of the three raters’ scores.

**Table 1:** Intraclass correlation coefficients among the three observers for ALPS indices

ICC	Left ALPS index		Right ALPS index	
	Absolute agreement	Consistency	Absolute agreement	Consistency
Single-measures	0.754 (95% CI: 0.629–0.849)	0.811 (95% CI: 0.708–0.887)	0.789 (95% CI: 0.677–0.872)	0.836 (95% CI: 0.744–0.902)
Average-meaures	0.902 (95% CI: 0.835–0.944)	0.928 (95% CI: 0.879–0.959)	0.918 (95% CI: 0.863–0.953)	0.939 (95% CI: 0.897–0.965)

*ICC * Intraclass correlation coefficient

### Behavioral performance

Three outliers were found (> 2SDs from the mean), who were excluded from the analysis. The assumption of sphericity was not met, as assessed by Mauchly’s test of sphericity, therefore an adjustment was made using Greenhouse and Geisser’s epsilon. The analysis of mean RT revealed a significant block main effect F(2.334, 93.343) = 33.963, *P* < 0.001, η^2^_p_ = 0.459. Post hoc analysis with a Bonferroni adjustment revealed significant difference in RTs between all blocks (adjusted P values ranged between 0.00000006 and 0.026) except between B3 and B4 (adjusted *P* = 0.089) and between B4 and B5 (adjusted *P* = 0.126).

### Associations between ALPS index and performance decrease

**Fig. 1:**
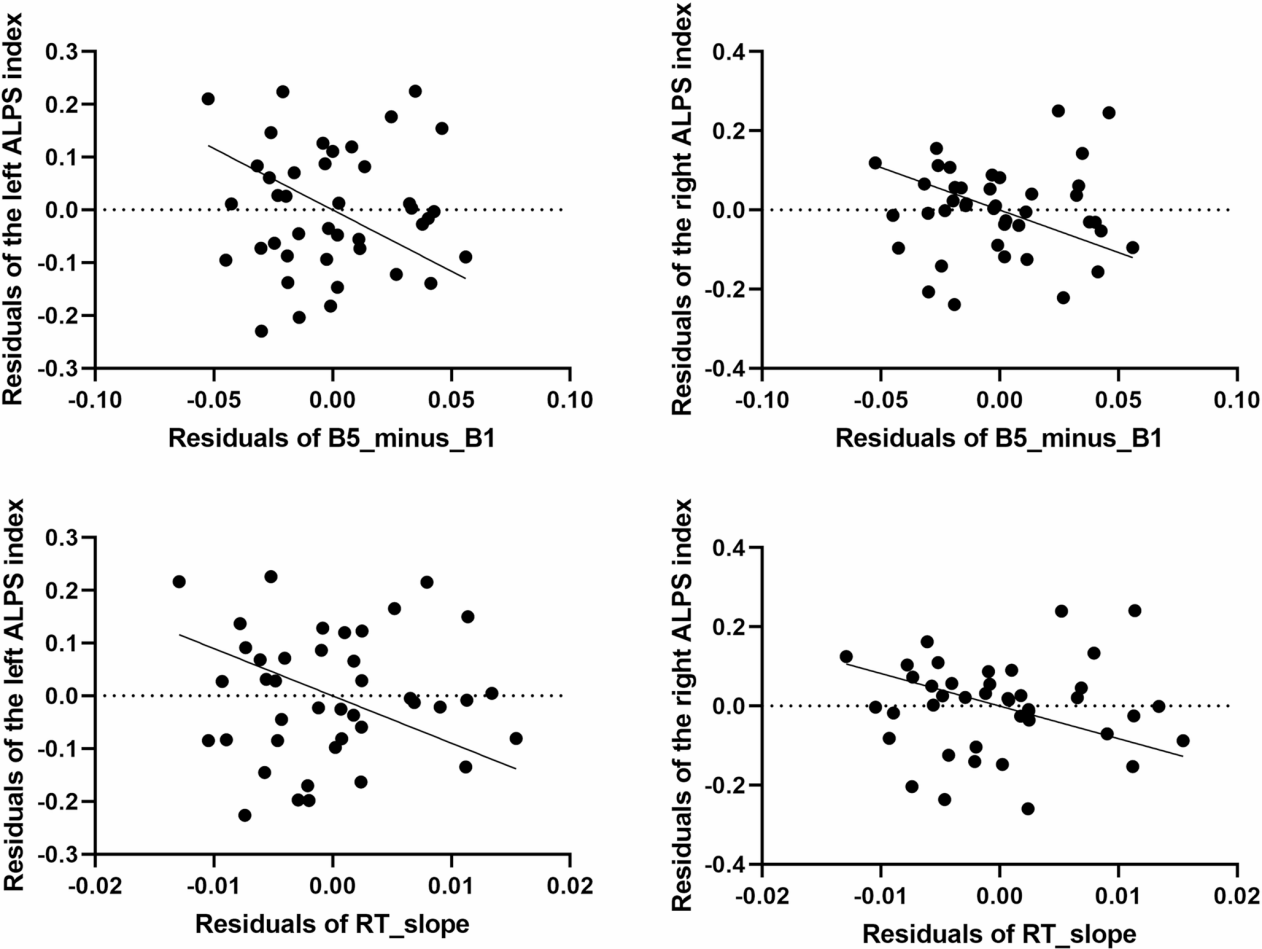
Partial regression plot showing the unique relationship between ALPS indices and reaction time changes, after controlling for age and sex. The plot displays the residuals of reaction time change (after removing the linear effects of age, sex and ESS) against the residuals of ALPS index (after removing the linear effects of age, sex and ESS). Each point represents an individual subject in the study. Negative slopes indicate that lower ALPS index values are associated with an increased reaction time decline during the task, independent of age, sex and ESS

One participant showed a greater studentized deleted residual than three SDs, therefore, we excluded the subject from the analysis. After controlling for the confounding effects of age, sex and ESS scores and using Benjamini-Hochberg correction to account for potential false discoveries a negative association was found between B5_minus_B1 and the left (b = −0.1, 95% CI [−0.16, −0.04]; *p* = 0.002) and right ALPS index (b = −0.1, 95% CI [−0.17, −0.04]; *p* = 0.003). Similar results were found when predicting RT_slope: both the left (b = −0.02, 95% CI [−0.04, −0.01]; *p* = 0.003) and right (b = −0.02, 95% CI [−0.04, −0.01]; *p* = 0.004) ALPS index showed negative associations (Fig. 1.). All four associations survived Benjamini-Hochberg correction for multiple comparisons. None of the covariates (age, sex and ESS scores) were associated with the dependent variables in either model.

## Discussion

The main finding of this study was that the left- and right-hemispheric ALPS indices were negatively associated with performance decrease during a psychomotor vigilance task, indicating that participants with low ALPS indices tended to show an increased performance decline during the task. These results are consistent with previous findings that investigated clinical cohorts and found positive associations between the ALPS index and cognitive performance. However, we can only speculate about possible mechanisms explaining the correlation between TOT effect and ALPS index.

Lower ALPS indices suggest slower water diffusion in the projection and association fibers for their respective hemispheres at the level of upper part of lateral ventricle body. Given the limited research on healthy individuals, extrapolating these results is challenging. To date, only three factors have been associated with the ALPS index in non-clinical populations: arterial pulsatility, aging, and sleep (see for a review (Yi et al., 2022). However, given the limited research on healthy individuals, not only is it challenging to extrapolate these findings, but it is also possible that additional factors have yet to be identified. Since arterial pulsatility has not been found to be related to cognitive performance and our sample comprised subjects within a narrow age range (and was also entered as a predictor to control for its influence), our interpretation focuses on sleep. Sleep has a critical function in ensuring the brain’s metabolic homeostasis. For example, Xie et al. (2013) found that natural sleep or anesthesia is associated with a 60% increase in the cortical interstitial space, resulting in an increased β-amyloid clearance rate. Thus, this function is particularly active during sleep, clearing potentially toxic waste substances that accumulate during wakefulness (Benveniste et al., 2019). Sleep deprivation studies further support this; for example, one night of total sleep deprivation impairs molecular clearance from the human brain, and subsequent sleep does not compensate for this impaired clearance (Stefani & Högl, 2021). A recent study revealed that ALPS index was negatively correlated with sleep quality in older but otherwise healthy adults (Ma et al., 2025). This study investigated functional and structural brain networks, and their findings added evidence that sleep quality affects cognitive health through the interaction between DTI ALPS and multimodal brain networks. Moreover, sleep deprivation has been associated with performance decline during prolonged cognitive tasks, and evidence indicates overlapping neural (Asplund & Chee, 2013) and genetic (Satterfield et al., 2017) backgrounds for sleep deprivation and TOT. The relationship between sleep quality and sustained attention performance in healthy young samples is also not new. Gobin et al. (2015) found a positive correlation between subjective sleep quality and performance on a ~ 25 min long sustained attention task in university students. It has also been shown that the sleep quality of university students is highly heterogeneous (> 30% report bad or very bad sleep quality) (Almojali et al., 2017). Thus, to summarize what has been described so far, we assume that there is a three-way association between ALPS index, sleep quality and sustained attention performance, however, the exact nature of these relationships remains to be clarified by future research. Moreover, these relationships can be observed in healthy young adults. Although it is important to emphasize that since this study was part of a larger research, we could not assess sleep quality, therefore our speculation is based on evidence from previous studies. Future studies with more sophisticated designs are needed to confirm this theory. Another problem is that ALPS index could also be influenced by contributions from axonal structures, rather than being exclusively tied to perivascular space diffusivity (Wright et al., 2024).

### Limitations and future directions

Notwithstanding the novelty of this study, the results should be interpreted with consideration of some limitations. First, although our sample reported adequate sleep quality the night before the assessment, the possible effect of sleep quality on the decline in performance related to mental fatigue should be considered when interpreting the results. Similarly, when interpreting our findings, we must consider how our subjects’ circadian rhythms may have affected the associations. Second, the sample size for regression analyses was small. Further studies with larger sample sizes and wider age ranges would lead to more reliable and generalizable findings. Although we used three observers and focused on both hemispheres to diminish major methodological challenges, DTI ALPS still has some significant adversities, such as different ROI sizes in the studies and indirect relationship between the DTI-ALPS index and human glymphatic function; thus our results should be interpreted with caution.

## Conclusions

In conclusion, higher diffusivity along the perivascular space, represented as an increased ALPS-index, was negatively associated with performance decrease during a prolonged exhaustive sustained attention task in both hemispheres. Sleepiness, age and sex did not affect these associations. Our results suggest that besides the clinical cohorts, ALPS index can be used as a marker of brain function in healthy individuals as well.

## Data Availability

The datasets generated during and/or analysed during the current study are available from the corresponding author on reasonable request.
